# The Middle Pleistocene human metatarsal from Sedia del Diavolo (Rome, Italy)

**DOI:** 10.1038/s41598-024-55045-1

**Published:** 2024-03-12

**Authors:** Alessandro Riga, Antonio Profico, Tommaso Mori, Riccardo Frittitta, Alessia Nava, Lucia Mancini, Diego Dreossi, Davorka Radovčić, Hannah Rice, Luca Bondioli, Damiano Marchi

**Affiliations:** 1 Department of Biology, University of Florence, Florence, Italy; 2 Department of Biology, University of Pisa, Pisa, Italy; 3 Department of Humanities, University of Ferrara, Ferrara, Italy; 4 Department of Odontostomatological and Maxillofacial Sciences, Sapienza University of Rome, Rome, Italy; 5 ZAG-Slovenian National Building and Civil Engineering Institute, Ljubliana, Slovenia; 6 Elettra-Sincrotrone Trieste S.C.P.A., Basovizza, Trieste, Italy; 7 Department of Geology and Paleontology, Croatian Natural History Museum, Zagreb, Croatia; 8 Department of Physical Performance, Norwegian School of Sport Sciences, Oslo, Norway; 9 Department of Cultural Heritage, University of Bologna, Bologna, Italy; 10 Service of Bioarchaeology, Museum of Civilizations, Rome, Italy; 11 Centre for the Exploration of Deep Human Journey, University of Witwatersrand, Johannesburg, South Africa

**Keywords:** Evolution, Anthropology, Palaeontology

## Abstract

The peopling of Europe during the Middle Pleistocene is a debated topic among paleoanthropologists. Some authors suggest the coexistence of multiple human lineages in this period, while others propose a single evolving lineage from *Homo heidelbergensis* to *Homo neanderthalensis*. The recent reassessment of the stratigraphy at the Sedia del Diavolo (SdD) site (Latium, Italy), now dated to the beginning of marine isotope stage (MIS) 8, calls for a revision of the human fossils from the site. In this paper, we present the morphometric, biomechanical and palaeopathological study of the second right metatarsal SdD2, to both re-evaluate its taxonomical affinities and possibly determine the levels of physical activity experienced by the individual during lifetime. Results demonstrate the persistence of archaic features in SdD2 suggesting new insights into the technology and hunting strategies adopted by *Homo* between MIS 9 and MIS 8.

## Introduction

Many of the evolutionary and ecological innovations in the genus *Homo* occurred in the Middle Pleistocene. Among them: (a) the emergence of *Homo sapiens* in Africa^1–3^ and *Homo neanderthalensis* in Europe^4,5^; (b) the emergence of prepared core technologies such as *Levallois*^6,7^; (c) the diffusion of the systematic use and control of fire^8–10^; and (d) the expansion of the distribution of our genus to high latitudes and altitudes^11^. The picture of how, when, and where these innovations developed is however still unclear, due to the complexity of these phenomena, the scarce fossil record, and gaps in the stratigraphy.

In Europe, hominin fossils dating to the Middle Pleistocene can be grouped in two main temporal clusters: before the beginning of marine isotope stage (MIS) 8 (~ 300 ka) and after the second half of MIS 7 (~ 200 ka). The latter is represented by Middle Pleistocene Neandertals such as the specimens from the sites of Altamura^12–14^, Krapina^15^, Saccopastore^16^, and La Chaise-de-Vouthon complex^17^. The most ancient cluster includes fossils dating from MIS 15 to MIS 9 and have an uncertain specific attribution. Some authors propose that all the Middle Pleistocene European fossils belong to the Neandertal lineage, thus they alternatively classify them as either *H. neanderthalensis*^5,18^ or *H. heidelbergensis* (intended as a chronospecies of Neandertals)^19–21^. Other authors propose that a Neandertal clade coexisted with another hominin clade represented by *H. heidelbergensis*^22–24^ or the recently proposed species *H. bodoensis*^25,26^. The last hypothesis is supported, according to its proponents, by the overlap in time of fossils with clear Neandertal affinities (e.g., Swanscombe and Sima de los Huesos)^27–29^ and European Middle Pleistocene fossils retaining archaic morphological traits (e.g., Mauer, Ceprano, Arago, and Aroeira)^29–32^.

European hominin fossils dating between ~ 300 and ~ 200 ka (MIS 8 and the early MIS 7) are poorly represented: some dental remains from Payre, dated to MIS 8–MIS 7^34,35^, attributed to *H. neanderthalensis*^36,37^; and the Apidima 1 partial cranium, dated to > 210 ka, showing a non-Neandertal morphology^38^. Filling this gap is of particular interest to palaeoanthropology because this period divides archaic Middle-Pleistocene humans, like *H. heidelbergensis*, and later humans confidently ascribable to Neandertals. At the same time, a fully *Levallois* technology emerges at the end of MIS 9 and the beginning of MIS 8^7^, although some authors propose a more ancient origin^6^.

In this framework, the recent reassessment of the stratigraphy at the archaeological and hominin-bearing site Sedia del Diavolo (SdD)^7,39^, assumes particular relevance. The site, until recently supposed to date at the late Middle Pleistocene^40,41^, is now dated at the beginning of MIS 8, between 295 and 290 ka^7^. This makes SdD among the few European sites with evidence of unambiguous *Levallois* technology between MIS 9 and early MIS 8 and probably the oldest from Italy^7^. Moreover, as far as we know, it represents the oldest association in Europe between hominin remains and *Levallois* technology.

**Figure 1:**
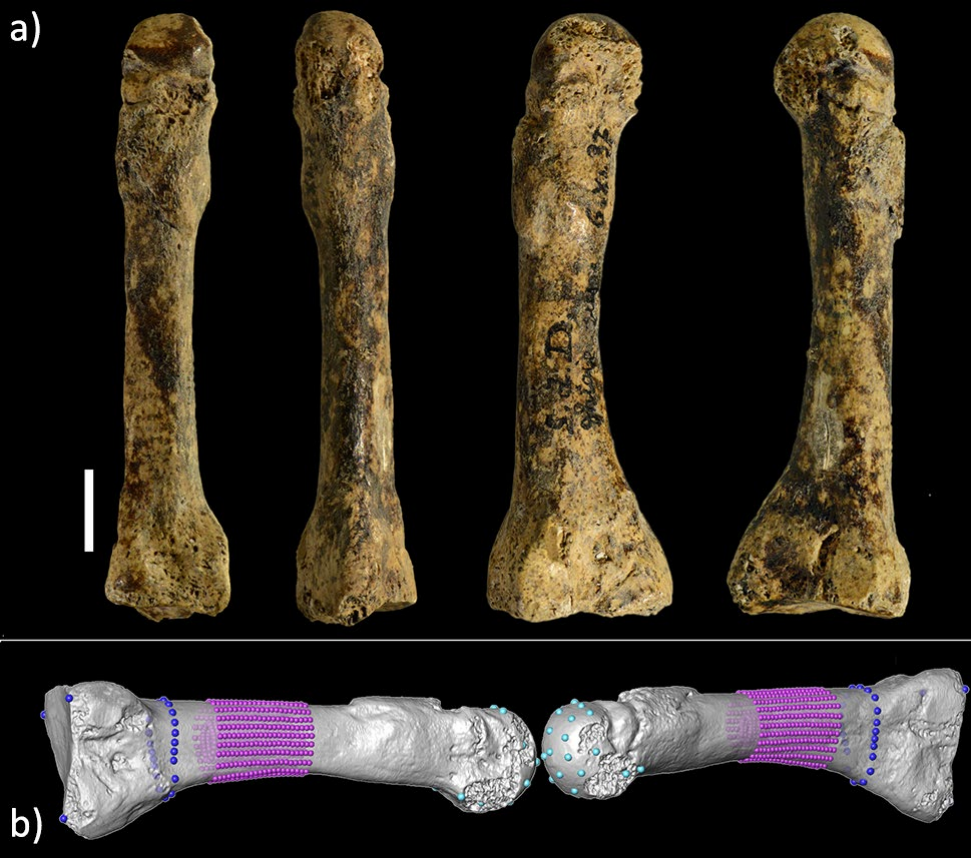
(**a**) the SdD2 fossil, a second right metatarsal with a bony callus on the distal diaphysis, interpreted as a stress fracture^38^. From left to right: dorsal, plantar, lateral, and medial views. Reference scale bar 10 mm. (**b**) the set of landmarks (large spheres) and semilandmarks (small spheres) used for the three-dimensional geometric morphometric analysis of the proximal epiphysis (dark blue), distal epiphysis (light blue) and diaphysis (purple).

Our analysis involves examining the morphology, biomechanical properties, and paleopathology of the human metatarsal from SdD (specimen SdD2, Fig. 1). The objective is to re-evaluate its taxonomical affinities and determine the levels of physical activity experienced by the individual during lifetime.

## Results

SdD2 (Fig. 1) is a virtually complete right second metatarsal. Its state of preservation is excellent and only minor fragments of cortical bone are lacking at the level of the inferior portion of the proximal surface, of the distal part of the superior lateral facet, and of some portions around the capitulum. Both the epiphyses are fused: in modern humans, the second metatarsal head fuses at 11–13 years in females and at 14–16 years in males^42^. The surface of the shaft is smooth, with no visible marks indicating the attachment points of the dorsal interossei muscle. Slight irregularities on the plantar aspect of the proximal epiphysis indicate the origin of the oblique head of the adductor hallucis muscle and the insertion of the tibialis posterior muscle.

### Bone stress injury

**Figure 2:**
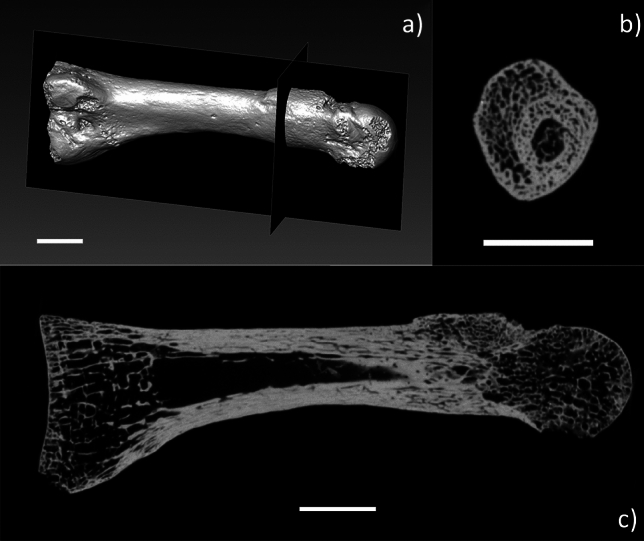
(**a**) Three-dimensional rendering obtained from the reconstructed microtomographic volume of SdD2, showing the location of the frontal (**b**) and sagittal (**c**) cross sections. Reference scale bar 10 mm. The images confirm the presence of a bony callus without misalignment of the diaphysis, a condition compatible with the presence of stress reaction.

In the distal third of the shaft, the contour of the cortical bone is characterized by a thickening of the bone due to the presence of a bone callus. Observing the inner structure in this area (Fig. 2; Supplementary Information Fig. S1) the bone thickening appears as a primary bone callus, not yet fully replaced by lamellar bone. The reconstruction of the three-dimensional (3D) microstructure (Fig. 2) shows that the diaphysis is well aligned and there is a weak endosteal reaction. This condition is compatible with a periosteal reaction caused by microfractures, rather than a compound or acute fracture, suggesting a diagnosis of stress fracture, or a ‘bone stress injury’ using updated terminology^43,44^. Furthermore, periarticular acute fractures are often associated with osteoarthritis^45,46^, while in SdD2, the distal articular surface shows no signs of bone proliferation or osteoarthrosis (Fig. 1a).

Other potential diagnoses, such as acute fracture of the bone, osteoma or osteomyelitis cannot be excluded, particularly without information related to the surrounding bones. However, there is no evidence in the internal structure of the bone (Supplementary Information Fig. S1) of a lesion or of circumferential sclerosis as could be expected in the case of osteoma or osteomyelitis, respectively^47^. Furthermore, the lack of any clear displacement of the bone along its long axis indicates a stress injury is more likely than an acute injury. Therefore, we consider a bone stress injury as the most probable diagnosis for the pathological condition observed in SdD2, in line with the diagnosis by a previous study^48^.

Metatarsals are one of the most common locations of stress injuries in active populations^49–51^, which are predominantly observed in the second and third metatarsals. Stress injuries of the second metatarsal bone typically occur in the distal shaft of the bone in the large majority of cases^52–54^.

### Cross-sectional geometry of the diaphysis

The cross-sectional geometrical properties of the distal portion of the diaphysis are influenced by the presence of the stress reaction (Supplementary Information Fig. S2). Nonetheless, moving proximally along the diaphysis from about 50% of the biomechanical length the effect of the stress reaction fades out and the geometric properties of SdD2 align with those of the comparative material. In our further analyses we considered only the portion between 50 and 70% of its biomechanical length, since proximally to 70% the geometrical properties seem to be unreliable (see Z_x_/Z_y_ plot in Supplementary Information Fig. S2), possibly due to problems in segmentation.

### Geometric morphometric analysis and relative cortical thickness of the diaphysis

**Figure 3:**
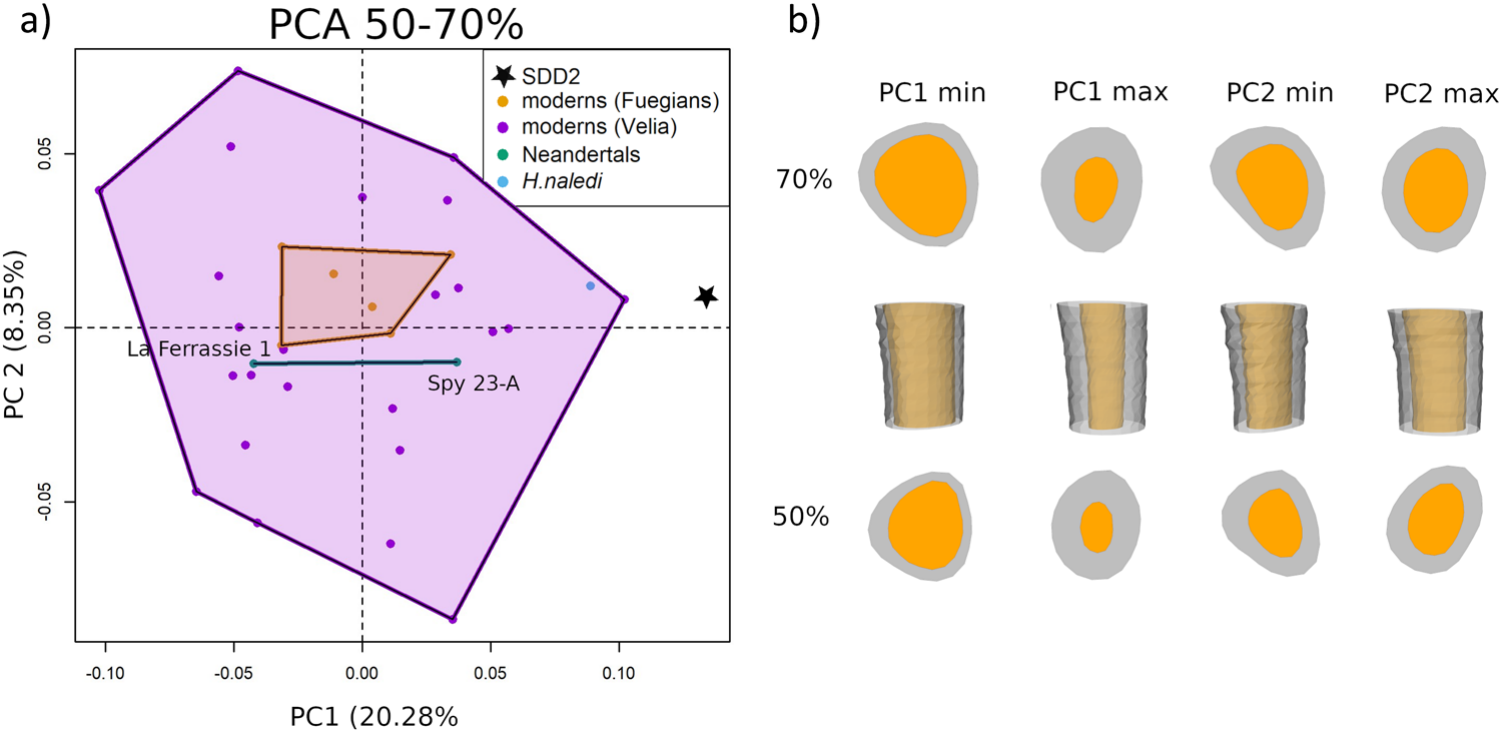
(**a**) Scatterplot of the first two principal components (PCs) from the geometric morphometric analysis of the diaphysis; (**b**) shape variation at the PCs extremes of the most proximal (70%) and most distal (50%) sections (proximal view) and of the whole portion of diaphysis analysed (lateral view).

We placed a set of n = 882 equally spaced semilandmarks as represented in Fig. 1b (purple spheres). We run a principal component analysis (PCA) (Fig. 3a) on the shape coordinates of the external and internal contours of the cortical bone after generalized Procrustes analysis on SdD2 and the comparative sample. Shape variations of the most proximal (70%) and the most distal (50%) sections are shown together with the shape variation of the whole portion of diaphysis analysed (Fig. 3b).

PC1 (which explains 20.28% of the total variance) is related to bone thickness and diaphyseal shape. Negative values correspond to cross sections with thinner cortex and more triangular shape, while positive values to cross sections with thicker cortex and more elliptical shape. SdD2 falls distinctly out of the variation of modern humans and Neandertals and quite far from *Homo naledi*, showing the thickest cortical bone in our sample (high PC1 values). The allometric signal for this PC is weak but statistically significant (see Supplementary Information S1).

PC2 (which explains 8.35% of the total variance) is related to the orientation of the major axis of the bone cross section. Negative values correspond to cross sections with a mediolaterally oriented major axis, while positive values to cross sections with a dorsoplantarly oriented major axis. The variability of modern humans encompasses that of the other groups. SdD2, as well as *H. naledi* and *H. neanderthalensis*, fall in the middle of the range of modern humans.

PC3 (which explains 8.33% of the total variance) is related to the distribution of the cortical bone with respect to the dorsoplantar and mediolateral diameters and orientation of major axis (see Supplementary Information Fig. S3). Positive values correspond to a more elliptical cross sections with a thinner cortex, while negative values correspond to a rounder cross sections with thicker cortical bone around the x and y axis. Modern human variation encompasses the entire range of variability. SdD2 and H. naledi fall at negative values in proximity of the mean shape.

**Figure 4:**
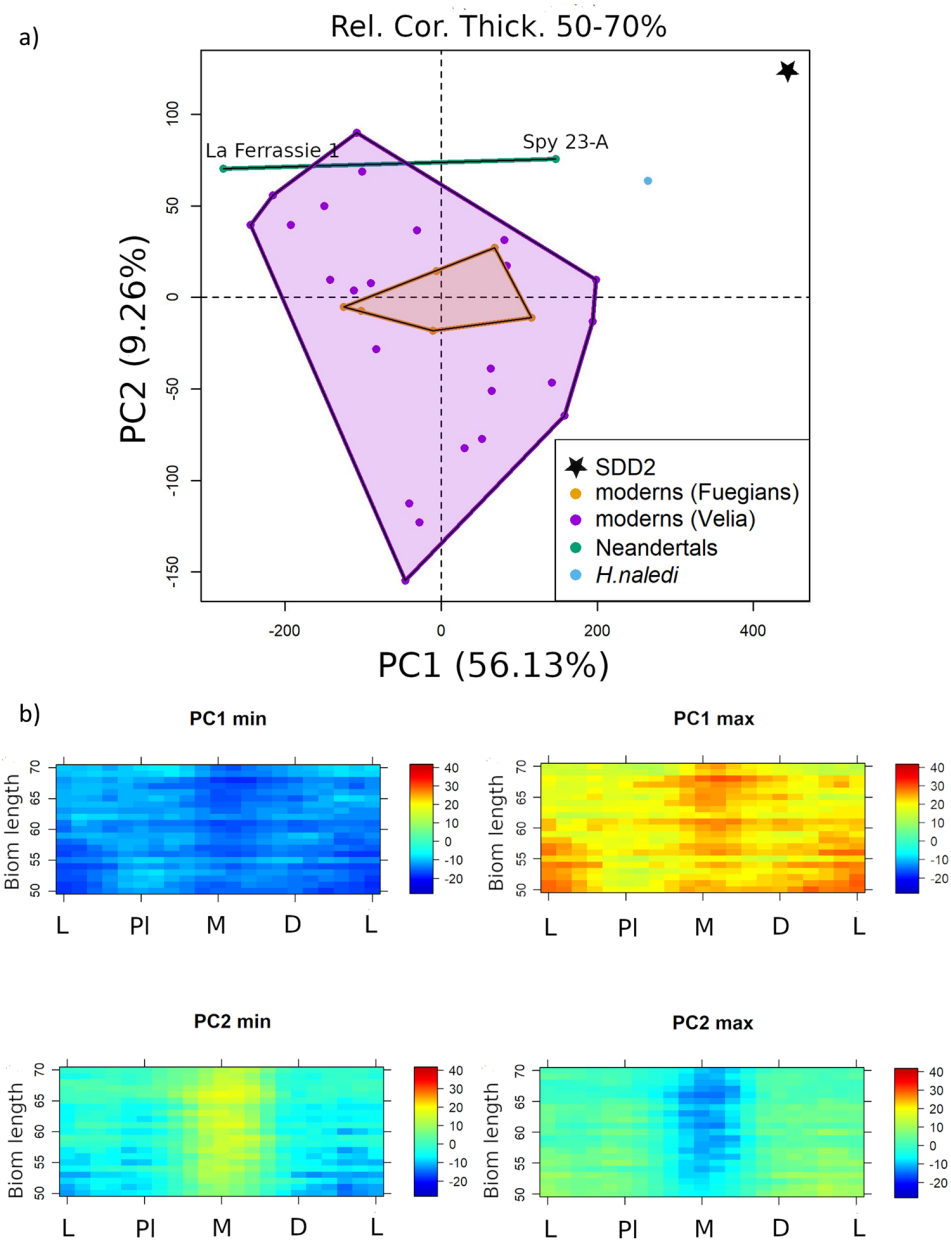
(**a**) scatterplot of the first two principal components (PCs) from the principal component analysis of the relative cortical thickness along the diaphysis (from 50 to 70% of biomechanical length); (**b**) colormap variations of relative cortical thickness.

The PCA on the colormaps of the relative cortical thickness values returns similar results (Fig. 4). The first three PCs account respectively for 56.13%, 9.26% and 4.15% of the total variance. PC1 is related to overall cortical thickness, with positive values corresponding to higher relative cortical thickness. SdD2, together with *H. naledi*, fall out of modern human variability for PC1 and has the highest PC1 value. Neandertals and modern humans show relatively lower cortical thickness and are widely overlapping.

PC2 is correlated with the distribution of cortical thickness around the longitudinal axis. Negative values correspond to thicker cortex medially, while positive values correspond to a thinner cortex in the medial aspect of the bone. SdD2 has the highest PC2 value and falls out of the modern human range. Neandertals and *H. naledi* are concentrated in the upper part of the modern human distribution, partially overlapping with them.

PC3 is associated with bone distribution from the proximal to the distal portion of the diaphysis. Negative values correspond to a thicker cortex distally, while positive values correspond to a thicker cortex proximally. SdD2, *Homo naledi* and La Ferrassie 1 fall in the range of modern humans. Spy 23A is at the opposite extremes of the distribution (see Supplementary Information Fig. S4).

### Proximal epiphysis

**Figure 5:**
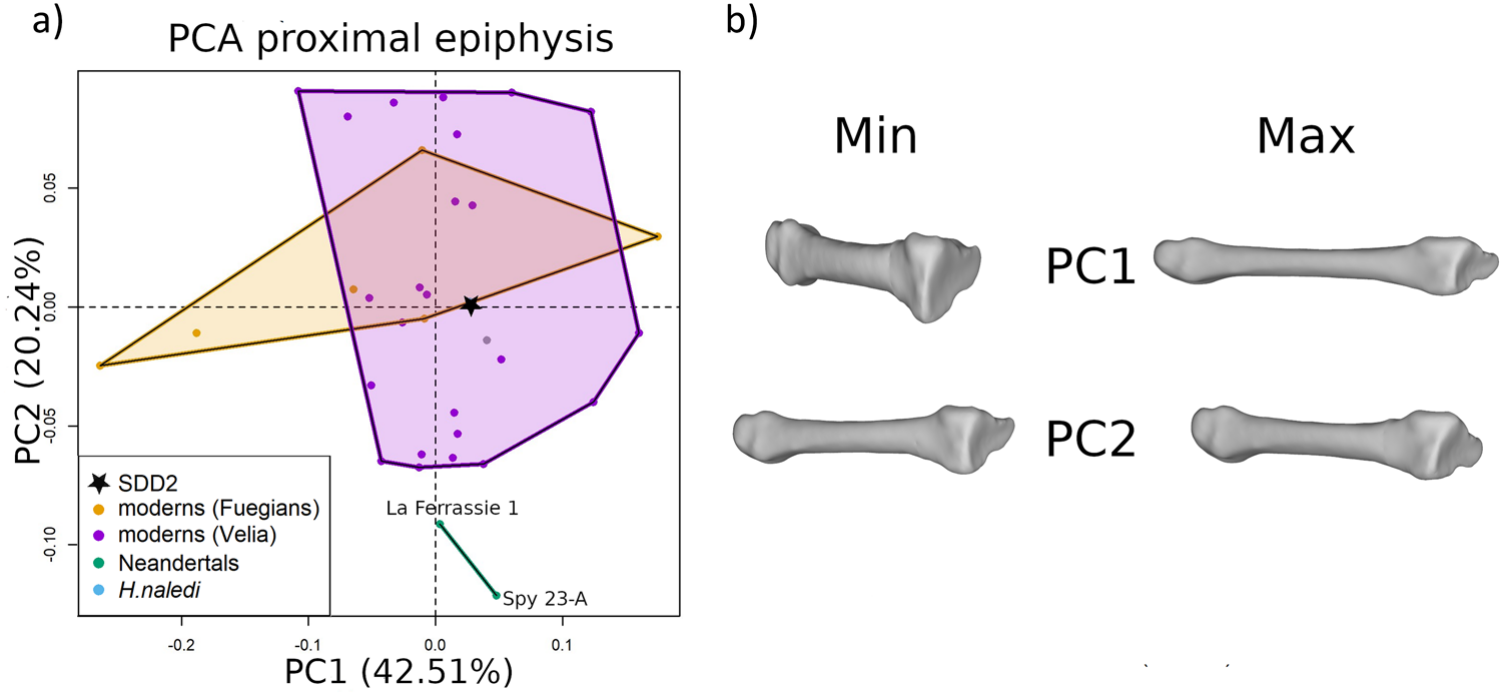
(**a**) scatterplot of the first two principal components (PCs) scores from the PCA of the shape analysis conducted on the proximal epiphysis; (**b**) shape variations of the whole metatarsal, obtained by warping the surface of SdD2 on the extreme values of the first two PCs scores.

We analysed the shape of the proximal epiphysis by placing three landmarks on the articular surface and a set of 21 semilandmarks on the external contour at the 80% of the biomechanical length (Fig. 1b, dark blue spheres). The results of the PCA and the shape variations are shown in Fig. 5 and Supplementary Information Fig. S5. PC1 (42.51% of the total variance) is related to the normal range of variability of the proximal epiphysis in modern humans and Neandertals. Interestingly, PC2 (20.24% of the total variance) distinguishes Neandertals from other modern humans and fossil specimens. While modern humans, *H. naledi* and SdD2 share a similar morphology, Neandertals are characterised by a rotation of the proximal epiphysis relative to the diaphysis.

### Distal epiphysis

**Figure 6:**
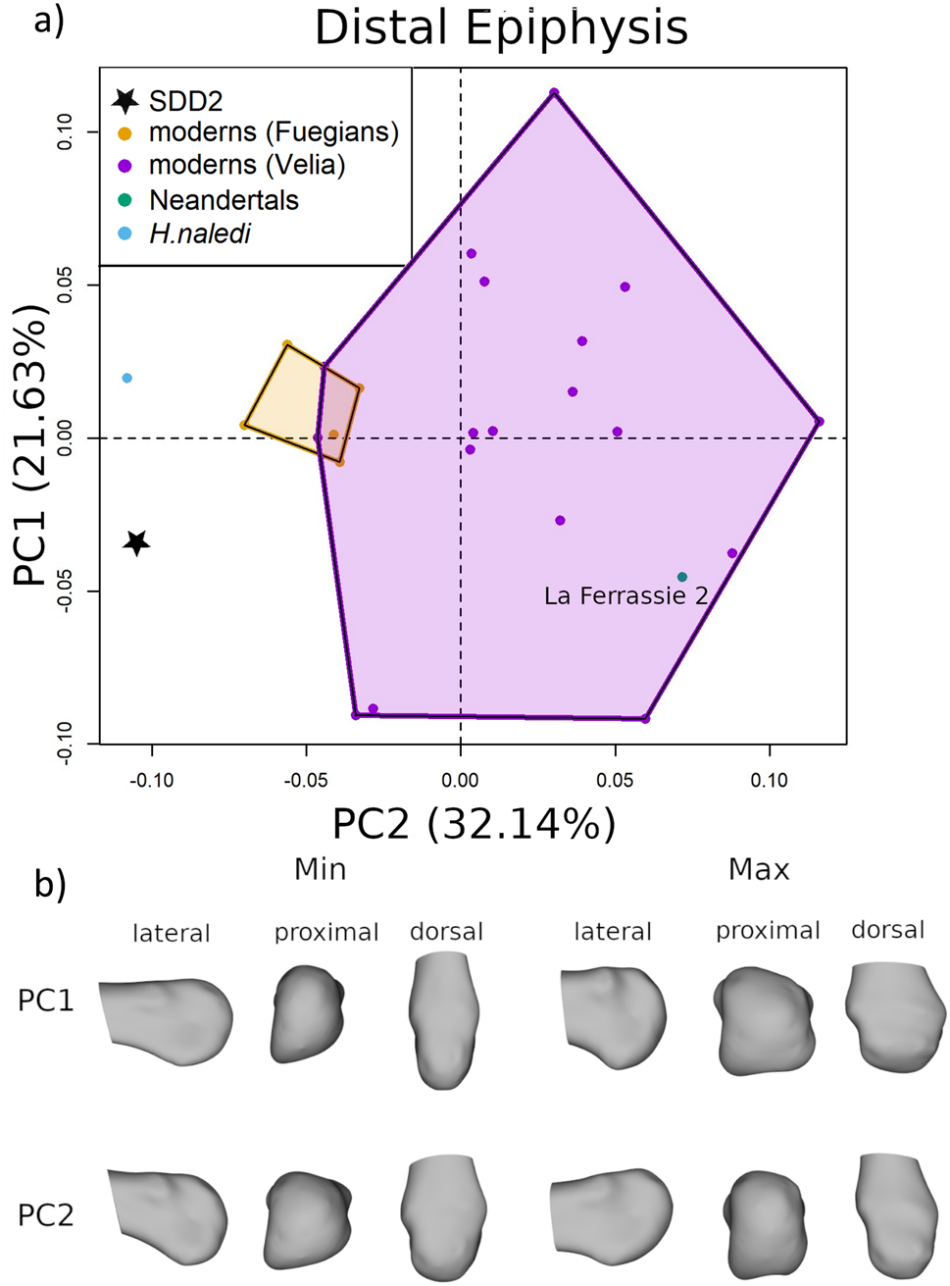
(**a**) scatterplot of the first two principal components (PCs) scores from the PCA of the shape analysis conducted on the distal epiphysis; (**b**) shape variations of the distal epiphysis metatarsal, obtained by warping the surface of SdD2 on the extreme values of the first two PCs scores.

We analysed the shape of the distal epiphysis by placing six landmarks on the articular surface and a set of 15 semilandmarks on the articular surface (Fig. 1b, light blue spheres). The results of the PCA and the shape variations are shown in Fig. 6 and Supplementary Information Fig. S6. The first three PCs account respectively for 32.14%, 21.63% and 13.24% of the total variance. Individuals with negative values of PC1 have a relatively proximodistally elongated and mediolaterally narrower metatarsal head, with a lateral contour rounder; the lateral plantar epicondyle is more developed than the medial plantar epicondyle. Individuals with positive PC1 values show a proximodistally less elongated and mediolaterally wider head, the medial plantar epicondyle more protruding plantarly and the dorsal lateral epicondyle more protruding dorsally. SdD2 and *H. naledi* fall at the negative extreme of PC1 partially overlapping with Fuegians, while Neandertals fall at the positive extreme. PC2 describes the orientation of the head and the expansion of the lateral plantar condyle. At the negative extreme, the head is slightly tilted dorsally, more square in contour and the lateral and medial plantar condyles are nearly equally developed; at the positive extreme, the head is slightly tilted plantarly, more rectangular in contour and the lateral plantar condyle is more developed. SdD2 and *H. naledi* fall in the negative range of modern humans, while Neandertals fall in the positive range of modern human distribution. PC3 is mostly related to the dorsal-plantar dimensions of the head, with negative values associated with dorsoplantarly narrower heads and positive values associated to dorsoplantarly wider heads. SdD2 and *H. naledi* fall within the variability of modern humans and have positive PC3 values, while La Ferrassie 2 (*Homo neanderthalensis*) is located at negative values (see Supplementary Information Fig. S6).

## Discussion

### Taxonomy and evolution

A previous study^48^ compared the second metatarsal Sedia del Diavolo 2 (SdD2) and other archaic and modern human fossil second metatarsals, using 13 linear measurements and indices. The author found some affinities between SdD2 and Neandertals, in agreement with the supposed chronology of the site in 1986. Our re-assessment, using biomechanical analysis and modern morphometric techniques, does not support this hypothesis. In all the analyses we conducted, SdD2 never clusters with Neandertals.

Concerning the diaphysis, Neandertals cluster with modern humans. SdD2, on the other hand, is characterised by an impressive thickness of the cortical bone that places it outside the variability of *H. sapiens*. To rule out the possibility that such an increase in cortical thickness was related to the stress reaction on the distal shaft, we analysed the geometrical properties of the shaft through CSG. Accordingly, we limited all other analyses to the portion of the bone with no pathological or apparent geometrical alterations. We are therefore confident that the extreme cortical thickness observed in SdD2 reflects the specimen’s normal, non-pathological, status. In this trait, SdD2 resembles other Middle Pleistocene *Homo* specimens, characterised by an extreme thickness in the postcranial cortical bone^55–58^ and parallel the extreme cortical thickness of the other hominin specimen found at Sedia del Diavolo (SdD1, central portion of a femoral diaphysis^48^).

Looking at the proximal epiphyseal shape, SdD2 falls, together with *H. naledi*, in the middle of modern human variability, while Neandertals are well separated along PC2. This points towards an ancestral morphology of the second metatarsal shared by SdD2, *H. sapiens* and *H. naledi*. Conversely, Neandertals have a derived morphology, characterised by a different orientation of the proximal epiphysis relative to the diaphysis. This is consistent with the hypothesis that Neandertal foot evolved some derived features, possibly related to functional or anatomical specialisation, as also suggested by other studies^59,60^. Also, the analysis of the distal epiphysis indicates differences between SdD2 and Neanderthals. SdD2 consistently falls on one side of the plots, next to modern human hunter-gatherers, while Neanderthals occupy the opposite extreme of the shape space (Fig. 6 and Supplementary Information Fig. S6). Previous studies^60^ did not observe major differences between Neanderthals and modern humans second metatarsals, possibly because they relied on linear measurements that cannot describe the relative position in the space between the diaphysis and epiphysis.

In summary, SdD2 has an archaic morphology, characterised by thick cortical bone and absence of some of the derived features typical of *H. neanderthalensis*. An archaic morphology lacking Neandertal-derived traits is seen, for other skeletal regions, in other Middle Pleistocene fossils such as Ceprano, and Mauer^29–31,33^, usually clustered into *H. heidelbergenis*. According to our interpretation, Sedia del Diavolo is among the latest evidence of fossils lacking Neandertal traits in the European Middle Pleistocene^29^, being dated to the beginning of MIS 8 (295–290 ka).

### Ecology and lifestyle

The human fossils from Sedia del Diavolo are associated with a clear *Levallois* technocomplex^7^. The new chronology, at the beginning of MIS 8^7,39^, makes Sedia del Diavolo the oldest hominin-bearing site yielding *Levallois*. *Levallois* (or Mode III) technology was widespread during the Middle Pleistocene in Africa and Eurasia. It is a defining feature of many Middle Palaeolithic and Middle Stone Age cultures^7,61^. In Europe, it is usually associated with Neandertals and in Africa with *H. sapiens*, though other species could have produced *Levallois* technocomplexes in eastern Asia^62^. Different hypotheses exist about the emergence of Levallois, implying either replacement of, or evolution from earlier lithic technologies^63^. Some authors suggested that the origin and spread of *Levallois* must be related to the dispersal of *H. heidelbergensis*^64^, while others proposed a multiregional evolution from previous lithic technologies^63,65^. Our analysis, which shows lack of affinities between SdD 2 and Neandertals, may suggest that European *Levallois* is not a prerogative of Neandertals and may therefore be associated in its early occurrences with other species, such as *H. heidelbergensis*. However, this does not necessarily mean a phylogenetic link between species and technologies. Indeed, in lithic technologies, phylogeny is a weak constraint, while mechanical, economic and ecological constraints are more important^66^.

Perhaps, the most intriguing clue into the lifestyle of Middle Pleistocene hominins is offered by the presence of a stress reaction on SdD2. In modern humans, the main cause of bony stress injuries is repetitive physical forces without adequate rest^54^ and these represent 3.7% of all sport-related injuries^52^. Metatarsal stress injuries are multifactorial and depend on biomechanical, anatomical, hormonal, and nutritional factors^67^. Pathological conditions affecting bone density, such as osteopenia and osteoporosis, increase the risk of developing stress injuries^68,69^. However, these conditions can be excluded from the microtomographic images of SdD2. On the other hand, training load is important, as indicated by the high prevalence of bony stress injuries in populations who undertake high volumes of weight-bearing activity (e.g.,^70,71^). Distance runners who train over 20 miles per week are particularly prone to metatarsal injuries^72,73^.

In the fossil record, we are aware of only two other metatarsal bone stress injuries, both on a fourth metatarsal. One is ATD6-124 from Gran Dolina^74^; the other is AT-534 from Sima de los Huesos^75^. All the metatarsal stress injuries available in the fossil record are dated between ~ 1 and 295 ka, during a period when, compared to later periods, the fossil record is relatively scarce.

This fact supports high physical activity levels, in particular walking and running, in the first representatives of the genus *Homo* in Europe. This is consistent with the endurance running hypothesis^76,77^. Following this hypothesis, endurance running had a central role in the evolution of our genus^76,78^. The capability of the members of the genus *Homo* of walking/running long distances may have helped in exploiting carcasses in the savannah in an early phase, and later allowed persistence hunting. Persistence hunting is a hunting technique still used by modern hunter-gatherers^78^, who run down prey chasing them during the hottest hours of the day, exploiting the human’s more efficient heat dissipation system (sweating vs. panting)^77^. Persistence hunting may be an explanation for the high prevalence of metatarsal stress injuries among the Early and Middle Pleistocene hominins and may indicate high levels of activity patterns of the species to which SdD2 belonged.

## Conclusion

Our re-assessment of the Sedia del Diavolo second metatarsal (SdD2) provides new evidenceon the evolution and ecology of *Homo* in Europe during the Middle Pleistocene. Though postcranial bones are not the ideal element for phylogenetic assessments, our analyses suggest that SdD2 does not belong to *Homo neanderthalensis*, a species characterised by a derived morphology of the second metatarsal. Instead, SdD2 exhibits a more archaic morphology with an extremely thick cortical bone. These observations, when interpreted within the context of the available fossil record, may suggest, the co-existence of at least two hominin clades in the Italian Peninsula during the beginning of marine isotope stage (MIS) 8.

Importantly, the Sedia del Diavolo site offers the oldest association of a hominin and *Levallois* technology, possibly challenging the long-held belief that only Neandertals produced *Levallois* in Europe. Additionally, the presence of bony stress injuries in the SdD2 specimen and their relatively high prevalence in the Early and Middle Pleistocene assemblages are consistent with the hypothesis of persistence hunting as a common technique among early members of the genus *Homo*.

## Materials

The comparative sample includes 34 right and left metatarsals from modern humans and fossil hominins. The modern human sample consists of 24 s metatarsals from the Imperial Roman rural site of Velia (1^st^ to second century AD), which are housed at the Museo delle Civiltà in Rome, and 6 s metatarsals belonging to Fuegian hunter-gatherers who died in the nineteenth century, which are housed at the Museum of Anthropology and Ethnology in Florence^79^. Fossil hominins include one right second metatarsal of *Homo naledi* (U.W. 101-1458)^80^ and three left second metatarsal of *Homo neanderthalensis* (La Ferrassie 1, La Ferrassie 2 and Spy 23-A)^81,82^.

## Methods

### Acquisition and reconstruction of X-ray microtomographic data

The SdD2 sample has been imaged by X-ray micro-computed tomography (microCT) using the FAITH instrument custom-developed at the Elettra synchrotron facility in Basovizza (Trieste, Italy). The instrument is based on a sealed microfocus X-ray source (Hamamatsu L12161-07) coupled, for this measurement, to a flat panel detector (Hamamatsu C11701DK-40, 2192 × 1776 pixels, 120 µm × 120 µm pixel size, active area of 265 mm × 215 mm). The parameters used for the imaged samples were: voltage = 110 kV, current = 106 µA, filter = 0.1 mm Cu, focal spot size = 20 µm, source-to-sample distance = 175 mm source-to-detector distance = 600 mm, exposure time per projection = 0.2 s, angular step = 0.2 degrees, total scan angle = 360 degrees. These settings corresponded to an effective pixel size of 35.0 × 35.0 µm^2^. Tomographic reconstructions were performed using the software Nrecon 1.7.0.4 (Bruker, USA) and an isotropic voxel size of 35 µm.

For the comparative sample, the *H. naledi* MT was CT scanned at the Johannesburg Hospital (Johannesburg, South Africa) on a Philips Brilliance 16P medical CT scanner (Philips Healthcare, Andover, MA); pixel dimensions 0.98 × 0.98 mm, voltage 120 kV. The Neandertal MT CT scans were CT scanned at the Muséum National d’Histoire Naturelle of Paris (France) on a Mercury medical CT scanner, pixel dimensions 0.185 × 0.185 mm, voltage 120 kV. The Fuegian material were CT scanned at the Santa Maria Nuova Hospital (Florence, Italy) on a Siemens Somatom medical CT scanner; pixel dimensions 0.416 × 0.416 mm, voltage 120 kV. The Velia MT CT scans were CT scanned at the Ospedale Santo Spirito (Rome, Italy) on a Philips Achieva D-Stream 1,5 T medical CT scanner, pixel dimensions 0.2135 × 0.2135 mm, voltage 120 kV.

### Bone orientation

We virtually oriented each metatarsal defining a set of *x*, *y*, *z* axes with the origin at the central point of the distal articular surface, the *x*-axis parallel to the dorsal surface of the distal epiphysis, the *y-*axis oriented toward the plantar surface, and the *z*-axis passing by the central points of the proximal articulation and the most distal point of the distal articulation, following previous works^83,84^. The bone biomechanical length has been calculated between the most extreme points laying on the *z*-axis^83,84^, and we set 0% and 100% to the distal and proximal ends, respectively, following the standard in CSG studies^85^. The Spy 23-A metatarsal was incomplete, lacking the distal epiphysis. We estimated its biomechanical length using La Ferrassie 1 as reference^86^. Since the comparative sample includes metatarsals of both sides, all left metatarsals have been mirrored.

### Cross-sectional geometry

We analysed the portion of the diaphysis from 20 to 80% of the biomechanical length. We extracted 61 cross Sects. (1% increments from 20 to 80%) and calculated cross-sectional geometric (CSG) properties using the R package “morphomap” vers. 1.2^87^. The CSG properties analysed are: percent of cortical area (CA%), polar section modulus (Z_p_) as an overall measure of diaphyseal strength, section modulus about the mediolateral axis (Z_x_) as dorsoplantar bending strength, section modulus about the dorsoplantar axis (Z_y_) as mediolateral bending strength, and the ratio Z_x_/Z_y_ as a shape index of the section^88^. We standardized the section modulus dividing it by [(mediolateral diameter × dorsoplantar diameter) × biomechanical length], following Ruff^88^.

### Geometric morphometrics

We defined three geometric morphometric datasets on the diaphysis and the proximal epiphysis (Fig. 1b). The first dataset is defined by 21 cross sections along the diaphysis between the 50% and 70% of the biomechanical length. At each cross section, we defined 21 equiangular semilandmarks on the external and on the internal outline of the cortical bone for a total of 882 semilandmarks. We performed generalized Procrustes analysis (GPA) translating, rotating and scaling the 35 semilandmark configurations (shape space). Subsequently, we performed a principal component analysis (PCA) on the shape variables, removing an outlier (Velia 113), and we calculated the shape variations along extreme range values of the first three principal component (PC) scores. The semilandmark configurations have not been slid since they are equiangular semilandmarks and not surface semilandmarks^86^.

The second dataset describes the shape of the proximal epiphysis and is defined by placing three anatomical landmarks at the three vertices of the articular facet and 21 equiangular semilandmarks on the outer surface of the diaphysis at the 80% of the biomechanical length. We performed GPA translating, rotating and scaling the 35 configurations (shape space). We analysed the aligned configurations by means of PCA. The configurations calculated at the extreme values of the first three PC scores have been used to create surface warping illustrating the shape variations.

The third dataset describes the shape of the distal epiphysis and is defined by six landmarks and 15 evenly-spaced semilandmarks on the articular surface. The six landmarks are type II landmarks described in a previous work^89^: (1) the most medially protruding point on the medial epicondyle; (2) the most plantarly projecting point on the medial plantar condyle; (3) the dorsal most point on the dorsal surface of the MT articular surface; (4) the most distally projecting point on the MT head; (5) the most laterally protruding point on the lateral epicondyle; (6) the most plantarly projecting point on the lateral plantar condyle. Spy 23A was excluded from the analysis because its distal epiphysis is missing. We performed GPA translating, rotating, and scaling the 34 configuration (shape space). The configurations calculated at the extreme values of the first three PC scores have been used to create surface warping illustrating the shape variations.

### Relative cortical thickness

We extracted 13 cross sections along the diaphysis between the 53% and 65% of the biomechanical length from each metatarsal. At each cross section, we defined a centre of gravity of the cross section and 21 paired equiangular semilandmarks on the external and on the internal outline of the cortical bone. We calculated the relative cortical thickness (*Rct*_*i*_) corresponding to each pair of semilandmarks (*i*) computing the ratio of the cortical bone thickness (*Ct*_*i*_) over the distance between centroid and outer surface (r_i_), ${Rct}_{i}=\frac{{Ct}_{i}}{{r}_{i}}$. The diaphysis is unrolled in a bi-dimensional matrix from the lateral margin towards the plantar direction [lateral (L) - plantar (Pl) - medial (M) - distal (D) - L]. Each matrix of cortical thickness is defined by 13 rows and 21 columns. The set of 35 matrices has been analysed by PCA, to evaluate variation in relative cortical thickness among samples. We calculated the relative cortical thickness at the extreme values of the first three PCs represented by colormaps.

### Supplementary Information

Supplementary Information.

## Data Availability

All data needed to evaluate the conclusions of the paper are present in the article and Supplementary Data S1. Data and R code are available at 10.5281/zenodo.10655437^90^.

## References

[1] Hublin JJ, Ben-Ncer A, Bailey S, Freidline SE, Neubauer S, Skinner MM, Bergmann I, Le Cabec A, Benazzi S, Harvati K, Gunz P (2017). New fossils from Jebel Irhoud, Morocco and the pan-African origin of *Homo sapiens*. Nature.

[2] Stringer, C. The origin and evolution of *Homo sapiens*. *Philos. Trans. R. Soc. Lond., B., Biol. Sci.* **371**, 20150237 (2016). 10.1098/rstb.2015.0237 PMC4920294 27298468

[3] Tattersall I (2009). Human origins: Out of Africa. Proc. Natl. Acad. Sci..

[4] Endicott P, Ho SYW, Stringer C (2010). Using genetic evidence to evaluate four palaeoanthropological hypotheses for the timing of Neanderthal and modern human origins. J. Hum. Evol..

[5] Hublin JJ (2009). The origin of Neandertals. Proc. Natl. Acad. Sci. U.S.A..

[6] Moncel MH, Ashton N, Arzarello M, Fontana F, Lamotte A, Scott B, Muttillo B, Berruti G, Nenzioni G, Tuffreau A, Peretto C (2020). Early Levallois core technology between Marine Isotope Stage 12 and 9 in Western Europe. J. Hum- Evol..

[7] Soriano S, Villa P (2017). Early Levallois and the beginning of the Middle Paleolithic in central Italy. PLoS one.

[8] Wrangham R (2017). Control of fire in the Paleolithic: Evaluating the cooking hypothesis. Curr. Anthropol..

[9] Sorensen AC (2017). On the relationship between climate and Neandertal fire use during the Last Glacial in south-west France. Quat. Int..

[10] Stahlschmidt MC, Miller CE, Ligouis B, Hambach U, Goldberg P, Berna F, Richter D, Urban B, Serangeli J, Conard NJ (2015). On the evidence for human use and control of fire at Schöningen. J. Hum. Evol..

[11] Fahu C, Welker F, Shen CC, Bailey SE, Bergmann I, Davis S, Xia H, Wang H, Fischer R, Freidline SE, Yu TL, Skinner MM, Stelzer S, Dong G, Fu Q, Dong G, Wang J, Zhang D, Hublin JJ (2019). A late middle Pleistocene Denisovan mandible from the Tibetan Plateau. Nature.

[12] Profico A, Buzi C, Di Vincenzo F, Boggioni M, Borsato A, Boschian G, Marchi D, Micheli M, Moggi-Cecchi J, Samadelli M, Tafuri MA, Arsuaga JL, Manzi G (2023). Virtual excavation and analysis of the early Neanderthal cranium from Altamura (Italy). Comm. Biol..

[13] Riga A, Boggioni M, Papini A, Buzi C, Profico A, Di Vincenzo F, Marchi D, Moggi-Cecchi J, Manzi G (2020). In situ observations on the dentition and oral cavity of the Neanderthal skeleton from Altamura (Italy). Plos one.

[14] Lari M, Di Vincenzo F, Borsato A, Ghirotto S, Micheli M, Balsamo C, Collina C, De Bellis G, Frisia S, Giacobini G, Gigli E, Hellstrom JC, Lannino A, Modi A, Pietrelli A, Pilli E, Profico A, Ramirez O, Rizzi E, Vai S, Venturo D, Piperno M, Lalueza-Fox C, Barbujani G, Caramelli D, Manzi G (2015). The Neanderthal in the karst: first dating, morphometric, and paleogenetic data on the fossil skeleton from Altamura (Italy). J. Hum. Evol..

[15] Rink W, Schwarcz H, Smith F, Radovĉiĉ J (1995). ESR ages for Krapina hominids. Nature.

[16] Marra F, Ceruleo P, Brian J, Pandolfi L, Petronio C, Leonardo S (2015). A new age within MIS 7 for the *Homo neanderthalensis* of Saccopastore in the glacio-eustatically forced sedimentary successions of the Aniene River Valley. Rome. Quat. Sci. Rev..

[17] Zanolli, C., Genochio, L., Tournepiche, J. F., Maurier, A. & Macchiarelli, R. The Neanderthal mandible BD 1 from La Chaise-de-Vouthon Abri Bourgeois-Delaunay (Charente, Southwestern France, OIS 5e). Dental tissue proportions, cortical bone distribution and endostructural asymmetry." *PALE*O*. Rev. Archéol. Préhist.* **30**, 346–359 (2020).

[18] Hublin, J. J. The Middle Pleistocene record: On the ancestry of Neandertals, modern humans and others. in A companion to paleoanthropology (ed. Begun, D. R.) 517–537 (John Wiley & Sons, 2013).

[19] Arsuaga JL, Martínez I, Gracia A, Lorenzo C (1997). The Sima de los Huesos Crania (Sierra de Atapuerca, Spain): A comparative study. J. Hum. Evol..

[20] Rosas, A. & Bermúdez de Castro, J. M. The mauer mandible and the evolutionary significance of *Homo heidelbergensis*. *Geobios* **31**, 687–697 (1998).

[21] Martinón-Torres, M., Bermúdez de Castro, J. M., Gómez-Robles, A., Bastir, M., Sarmiento, S., Muela, A. & Arsuaga, J. L. Gran Dolina-TD6 and Sima de los huesos dental samples: Preliminary approach to some dental characters of interest for phylogenetic studies. in *Dental Perspectives on Human Evolution: State of the Art Research in Dental Paleoanthropology *(eds. Bailey, S. & Hublin, J. J.) 65–79 (Springer, 2007).

[22] Tattersall, I. & Schwartz, J. H. The distinctiveness and systematic context of *Homo neanderthalensis*. in *Neanderthals revisited: New approaches and perspectives *(eds. Hublin, J. J., Harvati, K., Harrison, T.) 9–22 (Springer, 2006).

[23] Manzi G (2011). Before the emergence of *Homo sapiens*: Overview on the Early-to-Middle Pleistocene fossil record (with a proposal about *Homo heidelbergensis* at the subspecific level). Int. J. Evol. Biol..

[24] Profico, A., Di Vincenzo, F., Gagliardi, L., Piperno, M. & Manzi, G. Filling the gap. Human cranial remains from Gombore II (Melka Kunture, Ethiopia; ca. 850 ka) and the origin of *Homo heidelbergensis*. *J. Anthropol. Sci.* **94**, 1–24 (2016). 10.4436/JASS.94019 26583275

[25] Roksandic, M., Radović, P., Wu, X. J. & Bae, C. J. Resolving the “muddle in the middle”: The case for *Homo bodoensis* sp. nov. *Evol. Anthropol.* **31**, 20–29 (2022). 10.1002/evan.21929 PMC9297855 34710249

[26] Roksandic M, Radović P, Wu XJ, Bae CJ (2022). *Homo bodoensis* and why it matters. Evol. Anthropol..

[27] Stringer CB, Hublin JJ (1999). New age estimates for the Swanscombe hominid, and their significance for human evolution. J. Hum. Evol..

[28] Arsuaga J. L. Martínez, I., Arnold, L. J., Aranburu, A., Gracia-Téllez, A., Sharp, W. D., Quam, W. D., Falguères, C., Pantoja-Pérez, A., Bischoff, J., Poza-Rey, E., Parés, J. M., Carretero, J. M., Demuro, M., Lorenzo, C., Sala, N., Martinón- Torres, M., García, N., Alcázar de Velasco, A., Cuenca-Bescós, G., Gómez-Olivencia, A., Moreno, D., Pablos, A., Shen, C. C., Rodríguez, L., Ortega, A. I., García, R., Bonmatí, A., Bermúdez de Castro, J. M. & Carbonell, E. Neandertal roots: Cranial and chronological evidence from Sima de los Huesos. *Science* **344**, 1358–1363 (2014). 10.1126/science.1253958 24948730

[29] Roksandic M, Radović P, Lindal J (2018). Revising the hypodigm of Homo heidelbergensis: A view from the Eastern Mediterranean. Quat. Int..

[30] Mounier A, Marchal F, Condemi S (2009). Is Homo heidelbergensis a distinct species? New insight on the Mauer mandible. J. Hum. Evol..

[31] Di Vincenzo F, Profico A, Bernardini F, Cerroni V, Dreossi D, Schlager S, Zaio P, Benazzi S, Rubini M, Tuniz C, Manzi G (2017). Digital reconstruction of the Ceprano calvarium (Italy), and implications for its interpretation. Sci. Rep..

[32] Daura, J., Sanz, M., Arsuaga, J. L., Hoffman, D. L., Quam, R. M., Cruz Ortega, M., Santos, E., Gómez, S., Rubio, A., Villaescusa, L., Souto, P., Mauricio, J., Rodrigues, F., Ferreira, A., Godinho, P., Trinkaus, E. & Zilhão, J. New Middle Pleistocene hominin cranium from Gruta da Aroeira (Portugal). *Proc. Natl. Acad. Sci.* **114**, 3397–3402 (2017). 10.1073/pnas.1619040114 PMC5380066 28289213

[33] Bailey SE (2002). A closer look at neanderthal postcanine dental morphology: The mandibular dentition. Anat. Rec..

[34] Grün R, Aubert M, Joannes-Boyau R, Moncel MH (2008). High resolution analysis of uranium and thorium concentration as well as U-series isotope distributions in a Neanderthal tooth from Payre (Ardeche, France) using laser ablation ICP-MS. Geochim. Cosmochim. Acta.

[35] Valladas H, Mercier N, Ayliffe L, Falgueres C, Bahain JJ, Dolo JM, Froget L, Joron JL, Masaoudi H, Reyss JL, Moncel MH (2008). Chronology of the Middle Paleolithic sequence of Payre (Ardeche, France) based on radiometric dating methods. Quat. Geochronol. Quat. Sci. Rev..

[36] Condemi, S. Les restes humains. in *Le site de Payre. Occupations humaines de la moyenne vallée du Rhône de la fin du Pléistocène moyen et du début du Pléistocène supérieur* (ed Moncel, M. H.) 131–154 (Mémoire de la Société Préhistorique Française n°46, Paris, 2008).

[37] Verna C, Détroit F, Kupczik K, Arnaud J, Balzeau A, Grimaud-Hervé D, Bertrand S, Riou B, Moncel MH (2020). The Middle Pleistocene hominin mandible from ayre (Ardèche, France). J. Hum. Evol..

[38] Harvati K, Röding C, Bosman AM, Karakostis FA, Grün R, Stringer C, Karkanas P, Thompson NC, Koutoulidis V, Moulopoulos LA, Gorgoulis VG, Kouloukoussa M (2019). Apidima Cave fossils provide earliest evidence of *Homo sapiens* in Eurasia. Nature.

[39] Marra F, Ceruleo P, Pandolfi L, Petronio C, Rolfo MF, Salari L (2017). The aggradational successions of the Aniene River Valley in Rome: Age constraints to early Neanderthal presence in Europe. PloS one.

[40] Taschini M (1967). Il, “Protopontiniano” rissiano di Sedia del Diavolo e di Monte delle Gioie (Roma). Quaternaria..

[41] Caloi L, Palombo MR, Petronio CL (1980). fauna quaternaria di Sedia del Diavolo (Roma). Quaternaria.

[42] Cunningham, C., Scheuer L. & Black S. Developmental Juvenile Osteology. Second edition (Academic Press, 2016).

[43] Hoenig T, Ackerman KE, Beck BR, Bouxsein ML, Burr DB, Hollander K, Popp KL, Rolvien T, Tenforde AS, Warden SJ (2022). Bone stress injuries. Nat. Rev. Dis. Primers.

[44] Hoenig T, Tenforde AS, Hirschmüller A, Cassel M, Rolvien T, Hollander K (2023). Bone stress injuries. Dtsch. Z. Sportmed..

[45] Aguado-Maestro I, Panteli M, García-Alonso M, García-Cepeda I, Giannoudis PV (2017). Hip osteoarthritis as a predictor of the fracture pattern in proximal femur fractures. Injury.

[46] Anderson DD, Chubinskaya S, Guilak F, Martin JA, Oegema TR, Olson SA, Buckwalter JA (2011). Post-traumatic osteoarthritis: Improved understanding and opportunities for early intervention. J. Orthop. Res..

[47] Ortner, D. J. Identification of pathological conditions in human skeletal remains. Second Edition (Academic Press, 2003).

[48] Mallegni F (1986). Les restes humains du gisement de Sedia del Diavolo (Rome) remontant au Riss final. L’Anthropologie.

[49] Brukner P, Bradshaw C, Khan KM, White S, Crossley K (1996). Stress fractures: A review of 180 cases. Clin. J. Sport Med..

[50] Iwamoto J, Takeda T (2003). Stress fractures in athletes: Review of 196 cases. J. Orthop. Sci.

[51] Abbott A, Bird ML, Wild E, Brown SM, Stewart G, Mulcahey MK (2020). Part I: Epidemiology and risk factors for stress fractures in female athletes. Phys. Sportsmed..

[52] Chuckpaiwong B, Cook C, Pietrobon R, Nunley JA (2007). Second metatarsal stress fracture in sport: Comparative risk factors between proximal and non-proximal locations. Br. J. Sports Med..

[53] Hatch RL, Alsobrook JA, Clugston JR (2007). Diagnosis and management of metatarsal fractures. Am. Fam. Phys..

[54] Boden BP, Osbahr DC, Jimenez C (2001). Low-risk stress fractures. Am. J. Sports. Med..

[55] Trinkaus E, Ruff CB (2012). Femoral and tibial diaphyseal cross-sectional geometry in Pleistocene *Homo*. PaleoAnthropology.

[56] Ruff, C. B., Trinkaus, E., Walker, A. & Larsen, C. S. Postcranial robusticity in *Homo*. I: Temporal trends and mechanical interpretation. *Am. J. Phys. Anthropol.* **91**, 21–53 (1993). 10.1002/ajpa.1330910103 8512053

[57] Kennedy GE (1985). Bone thickness in Homo erectus. J. Hum. Evol..

[58] Rodríguez L, Carretero JM, García-González R, Arsuaga JL (2018). Cross-sectional properties of the lower limb long bones in the Middle Pleistocene Sima de los Huesos sample (Sierra de Atapuerca, Spain). J. Hum. Evol..

[59] Sorrentino R, Stephens NB, Marchi D, DeMars LJD, Figus C, Bortolini E, Bandino F, Saers JPP, Bettuzzi M, Boschin F, Capecchi G, Feletti F, Guarnieri T, May H, Morigi MP, Parr W, Ricci S, Ronchitelli A, Stock JS, Carlson KJ, Ryan TM, Belcastro MG, Benazzi S (2021). Unique foot posture in Neanderthals reflects their body mass and high mechanical stress. J. Hum. Evol..

[60] Pablos A, Gómez-Olivencia A, Maureille B, Holliday TW, Madelaine S, Trinkaus E, Couture-Veschambre C (2019). Neandertal foot remains from Regourdou 1 (Montignac-sur-Vézère, Dordogne, France). J. Hum. Evol..

[61] Tryon CA, McBrearty S, Texier PJ (2005). Levallois lithic technology from the Kapthurin formation, Kenya: Acheulian origin and Middle Stone Age diversity. Afr. Archaeol. Rev..

[62] Hu Y, Marwick B, Zhang JF, Rui X, Hou YM, Yue JP, Chen WR, Huang WW, Li B (2019). Late Middle Pleistocene Levallois stone-tool technology in southwest China. Nature.

[63] Arnaud J, Arzarello M, Lembo G, Muttillo B, Peretto C, Rufo E (2017). Between, “vintage” and “avant-guard”, the Lower Palaeolithic settlements in Molise region (Italy). Quat. Int..

[64] Foley, R. & Mirazón Lahr, M. Mode 3 technologies and the evolution of modern humans. *Camb. Archaeol. J.* **7**, 3–36 (1997).

[65] Adler DS, Wilkinson KN, Blockley S, Mark DF, Pinhasi R, Schmidt-Magee BA, Nahapetyan S, Mallol C, Berna F, Glauberman PJ, Raczynski-Henk Y, Wales N, Frahm E, Jöris O, MacLeod A, Smith VC, Cullen VL, Gasparian B (2014). Early Levallois technology and the Lower to Middle Paleolithic transition in the Southern Caucasus. Science.

[66] Brantingham PJ, Kuhn SL (2001). Constraints on Levallois core technology: a mathematical model. J. Archaeol. Sci..

[67] Pepper M, Akuthota V, McCarty EC (2006). The pathophysiology of stress fractures. Clin. Sports Med..

[68] Wilson DJ (2019). Osteoporosis and sport. Eur. J. Radiol..

[69] Marx RG, Saint-Phard D, Callahan LR, Chu J, Hannafin JA (2001). Stress fracture sites related to underlying bone health in athletic females. Clin. J. Sport Med..

[70] Sharma, J., Greeves, J. P, Byers, M., Bennett, A. N. & Spears, I.R. Musculoskeletal injuries in British Army recruits: A prospective study of diagnosis-specific incidence and rehabilitation times. *BMC Musculoskelet Disord*. **16**, 106 (2015). 10.1186/s12891-015-0558-6 PMC4443544 25935751

[71] Cosman F, Ruffing J, Zion M, Uhorchak J, Ralston S, Tendy S, McGuigan FEA, Lindsay R, Nieves J (2013). Determinants of stress fracture risk in United States Military Academy cadets. Bone.

[72] Sullivan D, Warren RF, Pavlov H, Kelman G (1984). Stress fractures in 51 runners. Clin. Orthop..

[73] Beddard, L., Roslee, C. & Kelsall, N. Acute and stress fractures of the metatarsals in athletes. Orthop. Trauma. In press.

[74] Martin‐Francés, L., Martinon-Torres, M., Gracia-Téllez, A. & Bermúdez de Castro, J., M. Evidence of stress fracture in a *Homo antecessor* metatarsal from Gran Dolina Site (Atapuerca, Spain). *Int. J. Osteoarchaeol.* **25**, 564–573 (2015).

[75] Gracia, A., Pablos, A., Martínez, I., Lorenzo, C., Carretero, J. M. & Arsuaga, J. L. Stress in a Middle Pleistocene hominid (Atapuerca, Spain): periosteal reaction compatible with fatigue fracture in a metatarsal bone. In *39th Annual Meeting of the Paleopathology Association, Portland (Oregon-USA)* (2012).

[76] Bramble DM, Lieberman DE (2004). Endurance running and the evolution of *Homo*. Nature.

[77] Carrier DR (1984). The energetic paradox of human running and hominid evolution. Curr. Anthropol..

[78] Liebenberg L (2006). Persistence hunting by modern hunter-gatherers. Curr. Anthropol..

[79] Moggi Cecchi, J. Le collezioni antropologiche. in *Il Museo di Storia Naturale dell'Università degli Studi di Firenze. Le collezioni antropologiche ed etnologiche/The Museum of Natural History of the University of Florence. The Anthropological and Ethnological Collections.* (eds. Moggi-Cecchi, J. & Stanyon, R.) 183–197 (University Press Firenze Firenze, 2014).

[80] Harcourt-Smith W, Throckmorton Z, Congdon K, Zipfel B, Deane AS, Drapeau MSM, Churchill SE, Berger L, DeSilva JM (2015). The foot of Homo naledi. Nat. Commun..

[81] Heim, J. L. *Les hommes fossiles de La Ferrassie II.* (Archives de l'Institut de Paléontologie Humaine 38, 1982).

[82] Berillon, G. Foot bones. in *Spy cave. State of 125 years of pluridisciplinary research on the Betche aux Rotches from Spy (Jemeppe-sur-Sambre, Province of Namur, Belgium), vol. 2*, (eds. Rougier, H., Semal, P. Royal Belgian Institute of Natural Sciences & NESPOS Society, 2014).

[83] Marchi D (2005). The cross-sectional geometry of the hand and foot bones of the Hominoidea and its relationship to locomotor behavior. J. Hum. Evol..

[84] Marchi D (2010). Articular to diaphyseal proportions of human and great ape metatarsals. Am. J. Phys. Anthropol..

[85] Ruff, C. B. & Hayes, W. C. Cross-sectional geometry of Pecos pueblo femora and tibiae—a biomechanical investigation: 1. Method and General Patterns of Variation. *Am. J. Phys. Anthropol.* **60**, 359–381 (1983). 10.1002/ajpa.1330600308 6846510

[86] Patel, B. A., Jashashvili, T., Bui, S. H., Carlson, K. J., Griffin, N., L., Wallace, I. J., Orr, C. M., & Susman, R. L. Inter-ray variation in metatarsal strength properties in humans and African apes: Implications for inferring bipedal biomechanics in the Olduvai Hominid 8 foot. *J. Hum. Evol.* **121**, 147–165 (2018). 10.1016/j.jhevol.2018.02.013 29764690

[87] Profico A, Bondioli L, Raia P, O'Higgins P, Marchi D (2021). Morphomap: An R package for long bone landmarking, cortical thickness, and cross-sectional geometry mapping. Am. J. Phys. Anthropol..

[88] Ruff, C. B. Biomechanical analyses of archaeological human skeletons. in *Biological anthropology of the human skeleton (3rd ed.)* (eds. Katzenberg, M. A. & Grauer, A. L.) 189–224 (John Wiley & Sons, 2019).

[89] Fernández PJ, Almécia S, Patel BA, Orr CM, Tocheri MW, Jungers WL (2015). Functional aspects of metatarsal head shape in humans, apes, and Old World monkeys. J. Hum. Evol..

[90] Profico A, Riga A (2024). Zenodo.

