# Supplementary material for: The Middle Pleistocene human metatarsal from Sedia del Diavolo (Rome, Italy)
Source: Sci Rep. 2024 Mar 12;14:6024. doi: 10.1038/s41598-024-55045-1 (PMC10933272; doi:10.1038/s41598-024-55045-1)
Supplement: Supplementary file 1 — Supplementary Information. [file 41598_2024_55045_MOESM1_ESM.docx]

**Supplementary Information**

**Supplementary Information Table 1**. List of the fossil and modern second metatarsals sample used in this study.

| **ID** | **Species** | **Site/population** | **Country** | **Side** | **Biomech. length (mm)** |
| --- | --- | --- | --- | --- | --- |
| SdD2 | N.I. | Sedia del Diavolo | Italy | R | 65.14 |
| U.W. 101-1458 | *H. naledi* | Rising star cave | South Africa | R | 61.93 |
| La Ferrassie 1 | *H. neanderthalensis* | La Ferrassie | France | L | 79.66 |
| La Ferrassie 2 | *H. neanderthalensis* | La Ferrassie | France | L | 69.35 |
| Spy 23-A | *H. neanderthalensis* | Spy | Belgium | L | 70.48 |
| Velia-51 | *H. sapiens* | Velia | Italy | R | 66.24 |
| Velia-57 | *H. sapiens* | Velia | Italy | R | 63.9 |
| Velia-66 | *H. sapiens* | Velia | Italy | R | 68.54 |
| Velia-70 | *H. sapiens* | Velia | Italy | L | 77.8 |
| Velia-71 | *H. sapiens* | Velia | Italy | L | 69.96 |
| Velia-75 | *H. sapiens* | Velia | Italy | R | 75.76 |
| Velia-78 | *H. sapiens* | Velia | Italy | L | 65.41 |
| Velia-80 | *H. sapiens* | Velia | Italy | R | 70.65 |
| Velia-82 | *H. sapiens* | Velia | Italy | L | 65.85 |
| Velia-83 | *H. sapiens* | Velia | Italy | R | 68.69 |
| Velia-86 | *H. sapiens* | Velia | Italy | R | 79.9 |
| Velia-90 | *H. sapiens* | Velia | Italy | L | 77.62 |
| Velia-94 | *H. sapiens* | Velia | Italy | R | 73.87 |
| Velia-99 | *H. sapiens* | Velia | Italy | L | 76.07 |
| Velia-100 | *H. sapiens* | Velia | Italy | R | 74 |
| Velia-113 | *H. sapiens* | Velia | Italy | R | 70.93 |
| Velia-131 | *H. sapiens* | Velia | Italy | R | 82.53 |
| Velia-134 | *H. sapiens* | Velia | Italy | L | 67.41 |
| Velia-137 | *H. sapiens* | Velia | Italy | L | 73.56 |
| Velia-140 | *H. sapiens* | Velia | Italy | L | 79.47 |
| Velia-146 | *H. sapiens* | Velia | Italy | L | 71.19 |
| Velia-154 | *H. sapiens* | Velia | Italy | R | 73.11 |
| Velia-181 | *H. sapiens* | Velia | Italy | R | 82.31 |
| Velia-194 | *H. sapiens* | Velia | Italy | L | 65.77 |
| Fueg_3116 | *H. sapiens* | Fuegians | Argentina | R | 60.11 |
| Fueg_3124 | *H. sapiens* | Fuegians | Argentina | L | 67.08 |
| Fueg_3241 | *H. sapiens* | Fuegians | Argentina | R | 70.69 |
| Fueg_3241-1 | *H. sapiens* | Fuegians | Argentina | R | 60.79 |
| Fueg_3241-2 | *H. sapiens* | Fuegians | Argentina | L | 65.88 |
| Fueg_3241-3 | *H. sapiens* | Fuegians | Argentina | L | 69.25 |

**
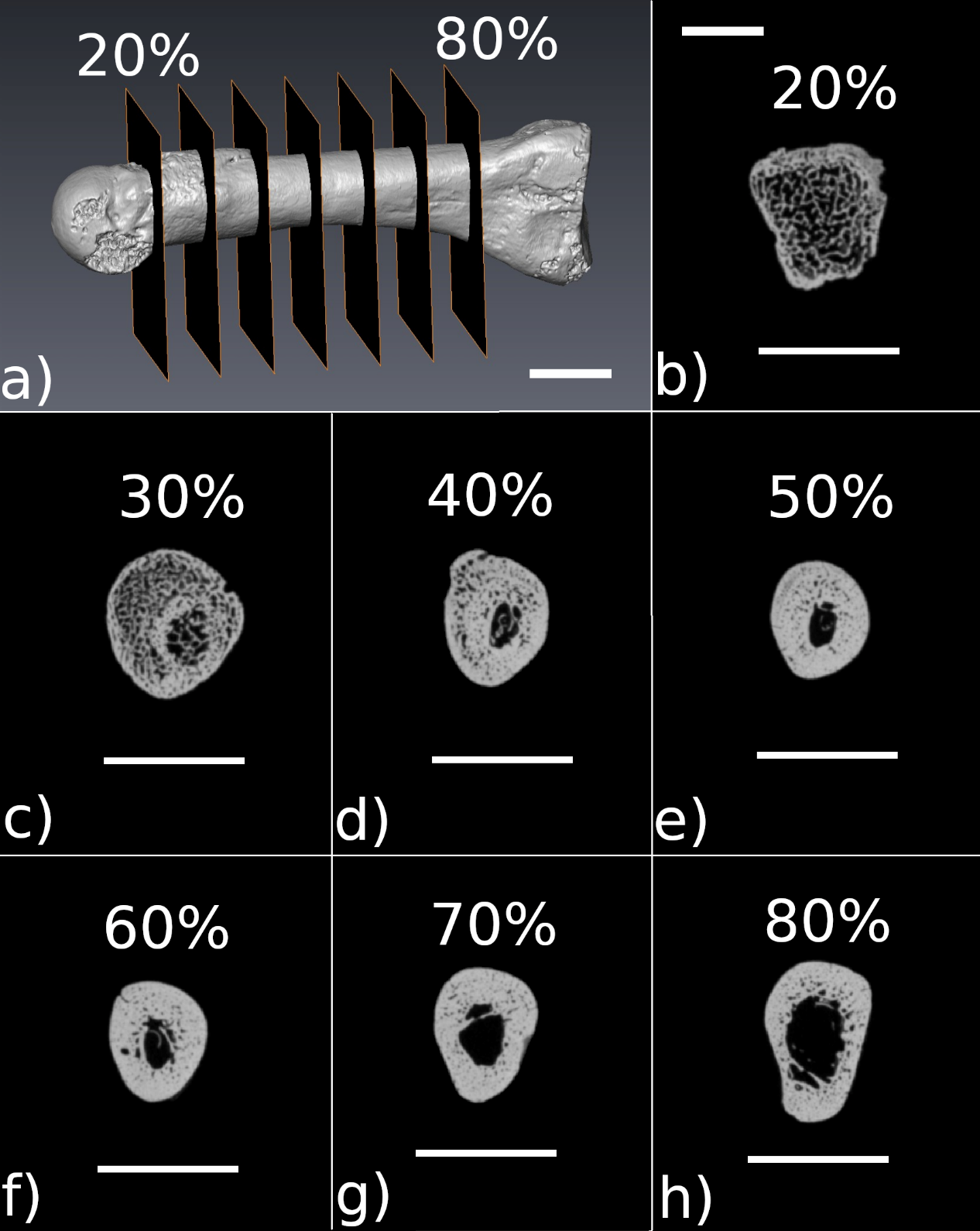
**

**Supplementary Information Fig. 1.** a) Three-dimensional model from the microtomographhic scan of the metatarsal SdD2, showing the longitudinal levels along the diaphysis of the cross sections shown in proximal view (b-h) from 20% (distal) to 80% (proximal) of the biomechanical length of the bone. Reference scale bars 10 mm.

**
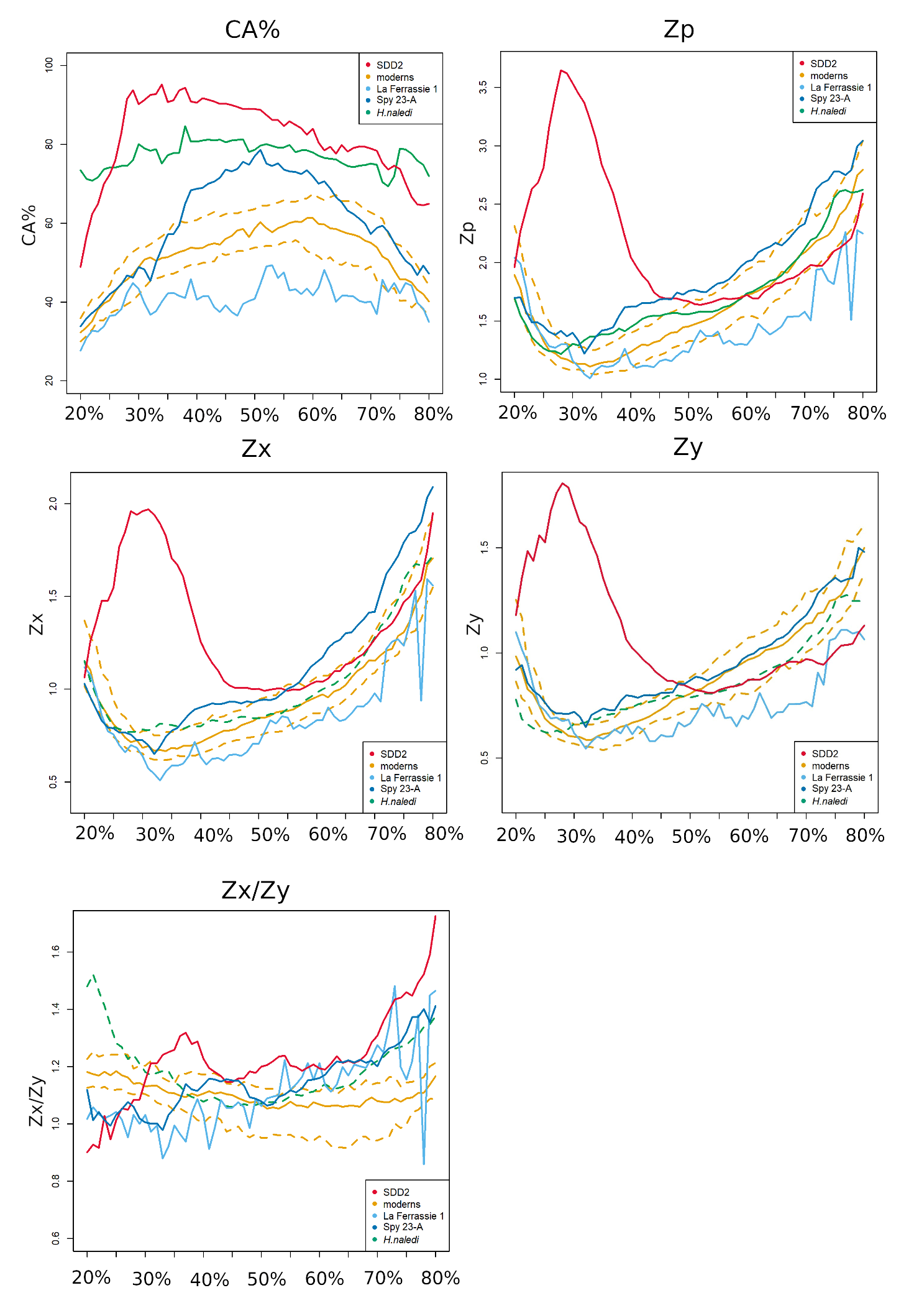
**

**Supplementary Information Fig. 2.** Cross-sectional geometric properties along the second metatarsal diaphysis from 20% (distal) to 80% (proximal) of the mechanical length of the bone. The presence of the stress fracture influences the appearance of cross sections in the distal portion of the bone. Nonetheless, proximally to ~ 50% the geometrical properties of SdD2 align to those of the comparative samples. CA = cortical area; Z_p_ = polar section modulus, torsional and average bending strength; Z_x_ section modulus about the mediolateral axis, dorsoplantar bending strength; = Z_y_ = section modulus about the dorsoplantar axis, mediolateral bending strength; Z_x_/Z_y_ = diaphyseal shape index.


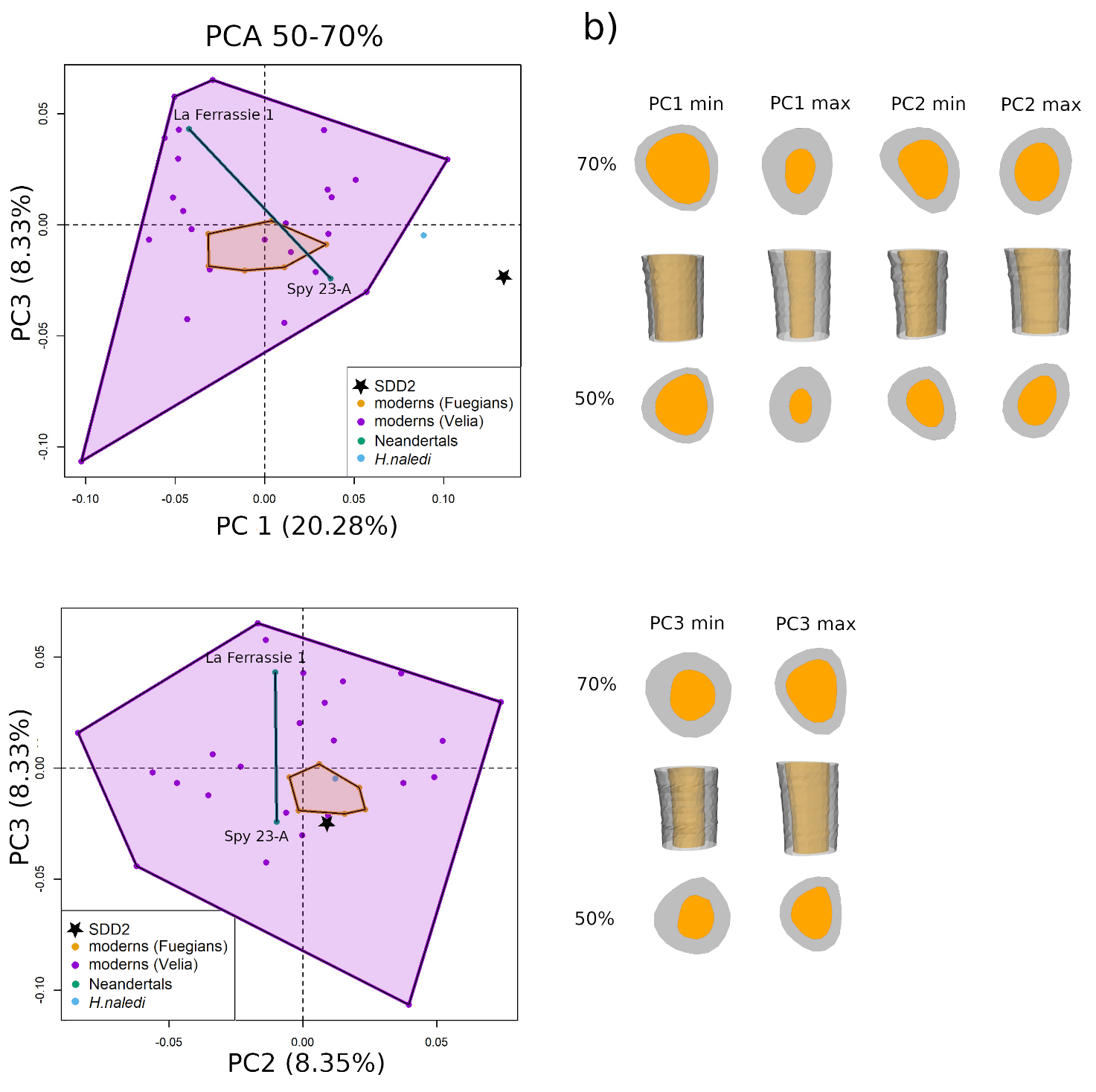


**Supplementary Information Fig. 3.** a) Scatterplots of the first three principal components (PCs) from the geometric morphometric analysis of the diaphysis; b) shape variation at the PCs extremes of the most proximal (70%) and most distal (50%) sections (proximal view) and of the whole portion of diaphysis analysed (lateral view).

**
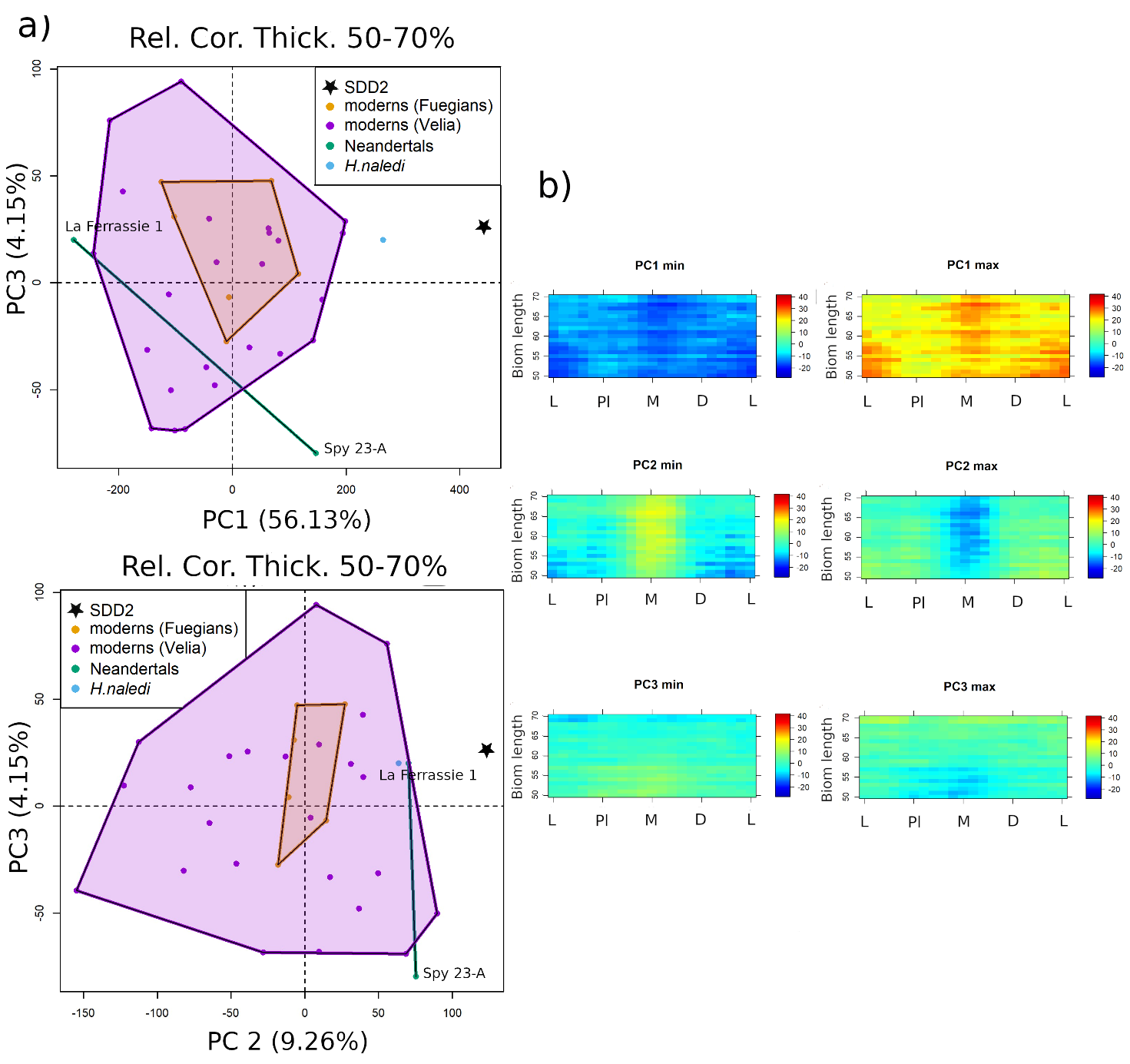
**

**Supplementary Information Fig. 4.** Fig. 4. a) additional scatterplots of the first three principal components (PCs) from the principal component analysis of the relative cortical thickness along the diaphysis (from 50% to 70% of biomechanical length); b) colormap variations of relative cortical thickness for the first three PCs.


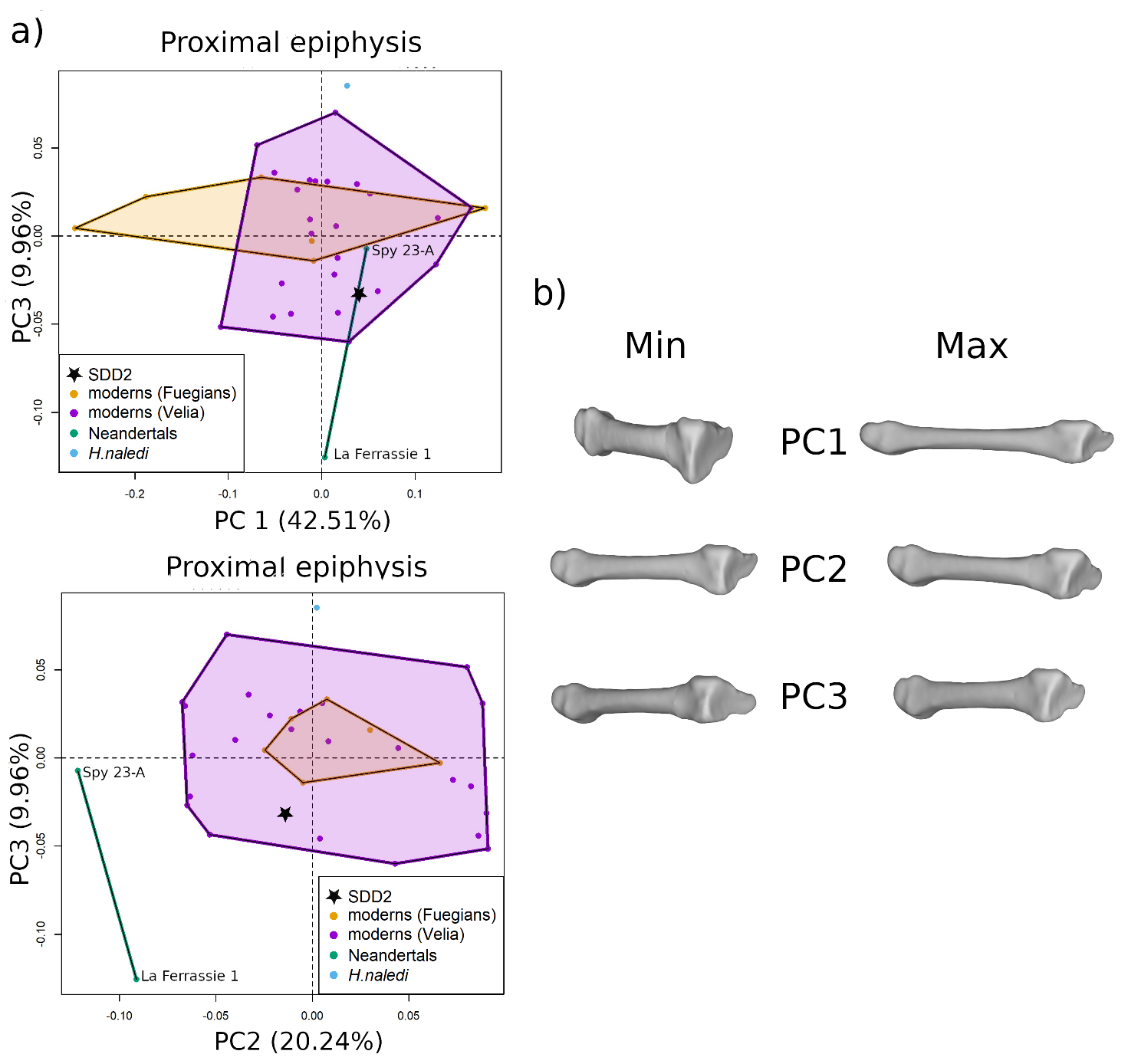


**Supplementary Information Fig. 5.** a) additional scatterplots of the first three principal components (PCs) scores from the PCA of the shape analysis conducted on the proximal epiphysis; b) shape variations of the whole metatarsal, obtained by warping the surface of SdD2 on the extreme values of the first three PCs scores.

**
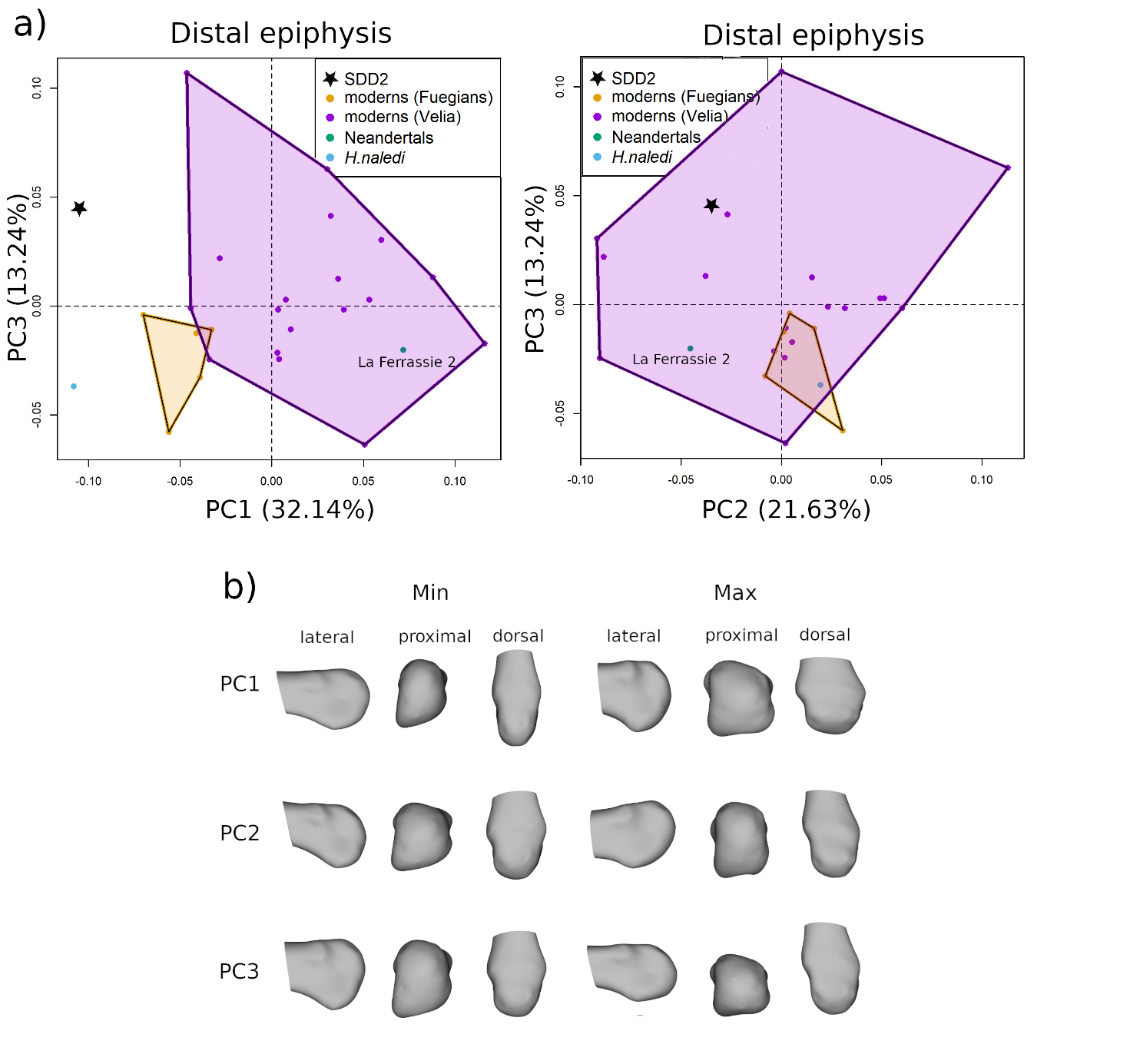
**

**Supplementary Information Fig. 6.** a) additional scatterplots of the first three principal components (PCs) scores from the PCA of the shape analysis conducted on the distal epiphysis; b) shape variations of the distal epiphysis metatarsal, obtained by warping the surface of SdD2 on the extreme values of the first three PCs scores.

**Allometry**

In the analysis of the diaphysis, using either geometric morphometrics (GM) or relative cortical thickness (RCT), the first principal component (PC) is related to cortical bone thickness. To understand if the variation in our sample is influenced by the size of the metatarsals, we performed a multivariate regression between the PCs and the biomechanical length of the bone. There is a weak signal for the entire sample (R^2^ = 0.051, *p* = 0.034 for GM; R^2^ = 0.094, *p* = 0.026 for RCT) and the PC1 (R^2^ = 0.138, *p* = 0.032 for GM; R^2^ = 0.152, *p* = 0.024 for RCT). In both cases it is a negative correlation (shorter metatarsals, thicker cortical bone). If we repeat the analysis only for the *Homo sapiens* group, the signal disappears. We conclude that there is not enough evidence to support allometry as an explanation of the differences in our sample.
